# Predicting linear B-cell epitopes using amino acid anchoring pair composition

**DOI:** 10.1186/s13040-015-0047-3

**Published:** 2015-04-29

**Authors:** Weike Shen, Yuan Cao, Lei Cha, Xufei Zhang, Xiaomin Ying, Wei Zhang, Kun Ge, Wuju Li, Li Zhong

**Affiliations:** 1 Department of Molecular Biology, Hebei University College of Life Sciences, 180 Wusi Road, Baoding, 071002 China; 2 Department of Laboratory Medicine General Hospital of Jinan Military Region, Jinan, Shandong 250031 China; 3 Center of Computational Biology, Beijing Institute of Basic Medical Sciences, Taiping Road 27, Beijing, 100850 China; 4 Centre Laboratory of Affiliated Hospital of Hebei University, Baoding, Hebei 071000 China; 5 Department of Basic Medical Sciences, Western University of Health Sciences, Pomona, CA 91766 USA

**Keywords:** Linear B-cell epitopes, Epitopes prediction, Amino acid anchoring pair composition

## Abstract

**Background:**

Accurate identification of linear B-cell epitopes plays an important role in peptide vaccine designs, immunodiagnosis, and antibody productions. Although several prediction methods have been reported, unsatisfied accuracy has limited the broad usages in linear B-cell epitope prediction. Therefore, developing a reliable model with significant improvement on prediction accuracy is highly desirable.

**Results:**

In this study, we developed a novel model for prediction of linear B-cell epitopes, APCpred, which was derived from the combination of amino acid anchoring pair composition (APC) and Support Vector Machine (SVM) methods. Systematic comparisons with the existing prediction models demonstrated that APCpred method significantly improved the prediction accuracy both in fivefold cross-validation of training datasets and in independent blind datasets. In the fivefold cross-validation test with Chen872 dataset at window size of 20, APCpred achieved AUC of 0.809 and accuracy of 72.94%, which was much more accurate than the existing models, e.g., Bayesb, Chen’s AAP methods and the enhanced combination method of AAP with five AP scales. For the fivefold cross-validation test with ABC16 dataset, APCpred achieved an improved AUC of 0.794 and A_CC_ of 73.00% at window size of 16, and attained an AUC of 0.748 and A_CC_ of 67.96% on Blind387 dataset after being trained with ABC16 dataset. Trained with Lbtope_Confirm dataset, APCpred achieved an increased Acc of 55.09% on FBC934 dataset. Within sequence window sizes from 12 to 20, APCpred final model on homology-reduced dataset achieved an optimal AUC of 0.748 and A_CC_ of 68.43% in fivefold cross-validation at the window size of 20.

**Conclusion:**

APCpred model demonstrated a significant improvement in predicting linear B-cell epitopes using the features of amino acid anchoring pair composition (APC). Based on our study, a webserver has been developed for on-line prediction of linear B-cell epitopes, which is a free access at: http:/ccb.bmi.ac.cn/APCpred/.

## Background

B-cell epitope is a part of an antigen recognized region that is bound to immunoglobulin molecules to stimulate B-cell response [1]. Based on structural characteristics, B-cell epitopes can be categorized into two types: linear (continuous) epitopes and conformational (discontinuous) epitopes. Linear epitopes are made up of short contiguous amino acids, whereas, conformational epitopes are composed of amino acids that are not contiguous in primary sequence but are brought together by the folded protein structure [2]. Although it is believed that the majorities of B-cell epitopes are discontinuous, detection of continuous epitopes still plays an important role in experimental designs, immunodiagnostic tests, and vaccines production [3,4]. However, development of a reliable computational method for predicting linear B-cell epitopes has been a daunting task with little success.

Previously, several studies have been conducted focusing on the correlations between physicochemical properties of certain amino acids and the linear B-cell epitopes within protein sequences. As a result, some epitope prediction methods have been constructed using physicochemical properties of amino acids, such as hydrophilicity [5], flexibility [6], turns [7], and solvent accessibility propensity scales [8]. These prediction models are simply based on the average of physicochemical values of amino acids at a window. However, these prediction models demonstrated only marginally better results than random selections [9]. Thus, new approaches should be developed to improve performance for prediction of linear B-cell epitopes.

Recently, some studies have attempted to improve the prediction accuracy using machine learning approaches. For example, the ABCpred [10] was developed using artificial neural network method. This model was constructed and evaluated using fivefold cross-validation tests on a training dataset, which was composed of a non-redundant dataset of 700 B-cell epitopes and 700 non-epitope peptides. Its input sequences ranged from 10 to 20 amino acids on the experimental design, and the best performance was achieved 65.93% prediction accuracy when ABCpred model was trained using recurrent neural network with a peptide dataset of 16 amino acids in length (ABC16). Then this model was further validated with a blind testing dataset (Blind387), and achieved 66.41% prediction accuracy.

Furthermore, Chen et al. [11] found that certain amino acid pairs (AAPs) tended to occur more frequently in B-cell epitopes, thus, an AAP propensity scale was used in combination with a support vector machine (SVM) to construct a prediction model, which reached an optimal accuracy of 71.09% on a dataset Chen872 containing 872 B-cell epitopes and 872 non-B-cell epitopes using fivefold cross-validation at window size of 20. Moreover, they combined the AAP scale and five amino acid propensity (AP) scales using the SVM classifier to improve the prediction accuracy, and the combination method achieved a better prediction accuracy of 72.54%. EL-Manzalawy et al. [12] reported an implemented AAP_BCPred_ method and developed a more superior model (BCpred) over those previous methods by utilizing SVM string kernels, and achieved the highest AUC (area under the receiver operating characteristic curve) of 0.758. In their results, BCpred and AAP_BCPred_ models both achieved improved prediction accuracies with fivefold cross-validation on ABC16 dataset, but attained lower prediction accuracy than ABCpred model when tested on blind dataset test [12]. Wee et al. [13] developed a SVM prediction model utilizing Bayes Feature Extraction – Bayesb. This Bayesb model achieved accuracy of 68.50% and AUC of 0.74 on testing with Chen’s dataset. Moreover, Singh et al. [14] recently reported an improved method called LBtope for linear B-cell epitope prediction using large datasets derived from immune Epitope Database. Testing performances of LBtope on some benchmark datasets still remained unsatisfactory.

In this study, we present a novel method APCpred for linear B-cell epitope prediction, which was derived from the combination of amino acid anchoring pair composition (APC) and Support Vector Machine (SVMs) methods using diverse lengths of peptides (12 to 20-mers). The performances of this model were evaluated using different public datasets.

## Methods

### Datasets

In order to develop prediction models, we collected six datasets (Table 1). The first dataset BCI727 was derived from the Bcipep database containing 2479 linear B-cell epitopes [15]. Each sample was a 20-mer peptide. If the epitope length was less than 20 amino acids, then the length was increased at both terminals by introducing equal number of residues derived from its original antigenic sequence [10]. If the epitope length was longer than 20 amino acids, the extra amino acids were removed at both terminals. In addition, we removed duplicated and highly homologous peptides by filtering the dataset based on 80% sequence identity using the CD-HIT program [16]. Furthermore, we obtained a dataset of 727 peptides (positive instances of B-cell epitopes) as positive samples. A total of 727 non-epitope peptides were generated by randomly extracting 20-mer peptide sequences from Swiss-Prot database while none of these negative instances occurred in the positive instances. This dataset was applied as the training dataset to develop our prediction model.

**Table 1:** **Six datasets for model construction and evaluation**

Dataset	PostiveNum*	NegativeNum*	Refereance
BCI727	727	727	Saha et.al. [10,15]
Chen872	872	872	Chen et.al. [11]
ABC16	700	700	Saha et.al. [10]
Blind387	187	200	Saha et.al. [10]
Lbtope_Confirm	1042	1795	Singh et.al. [14]
FBC934	934	934	EL-Manzalawy et.al. [17]

*PositiveNum and NegativeNum represent the number of positive samples and negative samples, respectively.

The second dataset, Chen872, was released by Chen [11], which contains 872 epitopes and 872 non-epitopes, and each of which was a 20-mer peptide. This dataset was used to evaluate our APCpred method in comparison with the Bayesb, Chen’s AAP and the combination method of AAP and AP in terms of fivefold cross-validation.

The third dataset, ABC16, was available from the model ABCpred, which contains 700 epitopes and 700 non-epitopes, and each of which was a 16-mer peptide [10]. This dataset was used to evaluate ABCpred in comparison with BCpred, AAP_BCpred_ and ABCpred in terms of fivefold cross-validation [12]. In addition, ABC16 was also used as training dataset for blind test in the next dataset Blind387 [10].

The fourth dataset, Blind387, was composed of 187 epitopes and 200 16-mer non-epitope peptides [10]. This dataset was used as a blind dataset to compare our model performance with the models BCpred, AAP _BCpred_ and ABCpred.

The fifth dataset, Lbtope_Confirm, was derived from IEDB by Singh [14]. This dataset contained variable lengths of 1042 unique B-cell epitopes and 1795 non-epitopes.

The sixth dataset, FBC934, was constructed by EL-Manzalawy [17]. The FBC934 contains 934 B-cell epitopes and 934 non-epitopes with variable lengths.

Among the datasets above, BCI727, Chen872 and ABC16 were applied to construct prediction models, which were evaluated by fivefold cross-validation. In addition, the dataset Blind387 was used as an independent dataset to test the performance of the models built from ABC16 dataset. Finally, both models APCpred and LBtope were developed using the dataset Lbtope_Confirm, and their performances were compared using the dataset FBC934.

### Feature extraction and machine learning method

**Figure 1:**
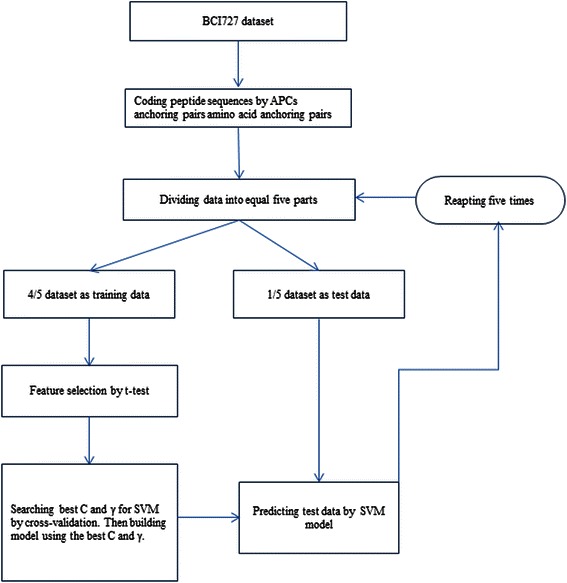
A schematic flowchart shows the feature selection and fivefold cross-validation test on BCI727 dataset.

To construct the prediction model of B-cell linear epitopes, amino acid anchoring pairs of short sequences were employed to represent the epitopes and non-epitopes. Feature selection was used to filter out the noise information on the sequence profile data. The prediction model was built and evaluated by fivefold cross-validation. During the validation, feature selection was made as part of cross-validation by employing 4/5 part of data to be feature-selected while leaving out 1/5 part of data as independent evaluation data that was not included in the feature selection. In addition, the completed model was built on the feature selection from a full training dataset by machine learning method, and was tested with an independent dataset. The evaluating design was shown in Figure 1. The performances of fivefold cross-validation were then compared among different methods.

### Amino acid anchoring pair composition

**Figure 2:**
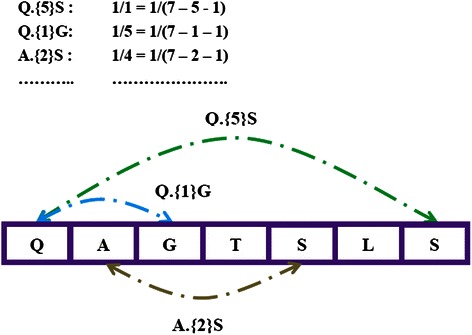
Amino acid anchoring pair composition extraction. For each sequence in a dataset, the sequences of amino acid anchoring pair composition (APC) are extracted by decomposing a protein or peptide sequences into 2-mer or *n*-mer subsequences. The two terminal amino acids of subsequences are the anchoring point pair that may anchor each other to form a relatively stable structure, and the pair composition can be used as the features of the peptide sequences.

For each sequence in a dataset, we extracted sequences of amino acid anchoring pair composition (APC) by decomposing a protein or peptide sequences into 2-mer or *n*-mer subsequences. We propose that the two terminal amino acids of subsequences are the anchoring point pair that may anchor each other to form a relatively stable structure, and the pair composition can be used as the features of the peptide sequences. For example, one sequence ‘QAGTSLS’ can be represented by the following features: ‘Q.{*0*}A’,‘A.{*0*}G’,’G.{*0*}T’…‘Q.{*1*}G’,’Q.{*2*}T’,…, ‘Q.{*5*}S’ . As shown in Figure 2, each feature was weighted by the frequency divided by the maximum likelihoods as this: one feature ‘*A*.{*i*}*A*’ (*A* denotes one of 20 amino acids, *i* denotes the number of interval amino acids and it is an integer) exits in one short sequence in number *k* and the window size of short sequence is a integer *l*, then the quantity of ‘*A.*{*i*}*A*’ in short sequence is calculated as *k*/(*l* – *i* - 1). For scanning all possible pairs, the number of interval amino acid pairs ranges from 0 to *I* (*I* denotes the max number of I, it is an integer) by step 1. Finally, there are 400*(I + 1) features describing each epitope sequence or non-epitope sequence. The setting of *I* is an important factor for prediction. To find the best *I*, we tested *I = 2, I = 3, I = 4* on BCI727 dataset at the window size of 20. The best parameter would be used on APCpred.

### Feature selection

Since there are many useless APC for discriminating epitopes from non-epitopes, we employed Student’s *t*-test to remove these noise APC without affecting on the classification of epitopes and non-epitopes. Cutoff p-value is an important factor to select features for model building. Traditional levels such as 0.05 would be a good cutoff value. However, in this study we first tried p < 0.05 to eliminate the non-discriminable anchoring pair compositions, but the prediction accuracies of APCpred resulted in poor AUCs. This might be due to the background noises of the dataset we used. Bursac et al. [18] and Budtz-Jørgensen et al. [19] used p-value cut-off point of 0.25 and 0.2 respectively in their studies and generated satisfied selection. Therefore, to find best *p*-value cutoff value, in this study we tried *p < 0.2, p < 0.4, p < 0.5, p <0.6* and *p = < 1* on the BCI727 at window size 20. The best parameter would be used on APCpred.

### Support vector machines and kernel methods

We applied support vector machines (SVMs) to construct prediction models. SVMs are a class of supervised machine learning methods used for classification and regression, and have been widely used in algorithm and modeling study [20]. Given a set of labeled training data (*x*_*i*_, *y*_i_), where *x*_i_ ∈ R^d^ and *y*_*i*_ ∈ {+1, −1}, training a SVM classifier involves finding a hyper-plane that maximizes the geometric margin between positive and negative training data samples. In this study, every component of the input vector *x* was the sequence anchoring pair occurring in the peptide.

When performing classification using the SVM classifier, because *x* is a combination of different features of a peptide, RBF (Radial Basis Function) kernel was used. The RBF is by far the most popular choice of kernel type used in SVM for its localization property. It is defined as [21]:

$$ K\left(x,x\hbox{'}\right)= \exp \left(-\lambda *{\left\Vert x-x\hbox{'}\right\Vert}^2\right) $$

The λ is a control parameter reflecting the kernel width.

For the RBF kernel, we found that tuning the SVM cost parameter C and the RBF kernel parameter γ were necessary and important to obtain satisfactory performances of SVM. We tuned these parameters using a two-dimensional grid searching method over the range C = 2^^-12^, 2^^-10^,…,2^^2^, γ = 2^^-5^, 2^^-3^,…2^^9^. It should be noted that the parameter optimization was performed only using the training data in inner-loop. The Lin Chih-Jen’s LIBSVM [22] was employed for both training and evaluation epitope prediction models.

### Fivefold cross-validation

In order to estimate parameters in unbiased manner in the feature extraction, a stratified fivefold cross-validation tests were applied (Shown as in Figure 1). Specifically, the sample dataset was randomly divided into five subsets, and each contained an equal number of peptides so that the relative proportion of epitopes to non-epitopes was 1:1. One fifth of the dataset was used as a testing dataset which was not used in the feature selection, while the feature selection was done and the learner was trained with the other four fifths dataset. This procedure was repeated five times, each time choosing different subsets of the data for training and testing. The whole consideration of the five testing sets was the final estimated performance of the training dataset.

### Performance evaluation

The threshold-dependent and threshold-independent measures were used to evaluate the performance of fivefold cross-validation on training and independent testing datasets. For threshold-dependent measures, we used four types of commonly used parameters to evaluate the performances of prediction algorithms in the experiment, the prediction accuracy (A_CC_), sensitivity (S_en_), specificity (S_pe_), and Matthews correlation coefficient (M_CC_). The M_CC_ measure has a value in the range from −1 to +1, and the closer the value to +1, the better the predictor is. A_CC_, S_en_, S_pe_, and M_CC_ are defined as follows:

$$ {\mathbf{A}}_{\mathbf{CC}} = \left(\mathbf{T}\mathbf{P}+\mathbf{T}\mathbf{N}\right)/\left(\mathbf{T}\mathbf{P}+\mathbf{F}\mathbf{P}+\mathbf{T}\mathbf{N}+\mathbf{F}\mathbf{N}\right) $$

$$ {\mathbf{S}}_{\mathbf{en}} = \mathbf{T}\mathbf{P}/\left(\mathbf{T}\mathbf{P}+\mathbf{F}\mathbf{N}\right) $$

$$ {\mathbf{S}}_{\mathbf{pe}}=\mathbf{T}\mathbf{N}/\left(\mathbf{T}\mathbf{N}+\mathbf{F}\mathbf{P}\right) $$

$$ {\mathbf{M}}_{\mathbf{CC}} = \left(\mathbf{T}\mathbf{P}*\mathbf{T}\mathbf{N}\ \hbox{--}\ \mathbf{F}\mathbf{P}\ *\mathbf{F}\mathbf{N}\right)/\mathbf{sqrt}\left(\left(\mathbf{T}\mathbf{N}+\mathbf{F}\mathbf{N}\right)\left(\mathbf{T}\mathbf{N}+\mathbf{F}\mathbf{P}\right)\left(\mathbf{T}\mathbf{P}+\mathbf{F}\mathbf{N}\right)\left(\mathbf{T}\mathbf{P}+\mathbf{F}\mathbf{P}\right)\right) $$

TP, FP, TN, and FN are abbreviated for the number of true positive sample, false positive sample, true negative sample, and false negative sample, respectively.

Threshold-dependent measures are likely to increase the number of true positives of the classifier at the expense of increasing in false positive, and they are often employed to access the performances of machine learning methods. However, threshold-dependent measures are difficult to access the overall performance of B-cell linear epitopes prediction. Receiver operating characteristic (ROC) curves can define the performance of a classifier for a threshold-independent method over all possible thresholds. Area under curve (AUC) measures discrimination ability of correctly classifying B-cell linear epitopes and non-epitopes. Any classifier performing better than random will have an AUC value that lies between 0.5 and 1.

## Results

### Identification of optimal parameters

AUC value was used to find the optimal combinations of parameters. For each combination of *I* (=2, 3, 4) and *p* (<0.2, 0.4, 0.5, 0.6, 1) on BCI727 dataset at window size 20, AUC value was calculated by fivefold cross-validation. The epitope and non-epitope sequence features were generated from the peptide sequence using APC at window size of 20. Then, we removed the noise APC by feature selection using *t*-test. The dimensional reduced APC were used for SVMs trainings and model evaluation. The results of a serial AUC values were shown in Table 2. The results indicated that *I = 3* setting had greater AUC values than those *I = 2* and *I = 4* settings, and the differences were statistically significant (Wilcoxon test, *p*-values were 0.02895 and 0.03125 respectively). While for *I* = 3, the results illustrated that the optimal *p*-value was at *p* < 0.5. Therefore, the optimal parameters for APCpred model development were *I* = 3 and *p* < 0.5.

**Table 2:** **AUC values for each combination of** ***I*** **(=2, 3, 4) and** ***p*** **(<0.2, 0.4, 0.5, 0.6, 1) on BCI727 dataset at window size of 20**

Parameters	*p* < 0.2	*p* < 0.4	*p* < 0.5	*p* < 0.6	*p* = < 1
*I* = 2	0.703	0.723	0.747	0.734	0.745
*I* = 3	0.715	0.742	0.748*	0.738	0.746
*I* = 4	0.704	0.726	0.725	0.723	0.730

* The bold denotes the largest AUC value of the prediction.

### Construction of prediction model for B-cell epitopes

**Figure 3:**
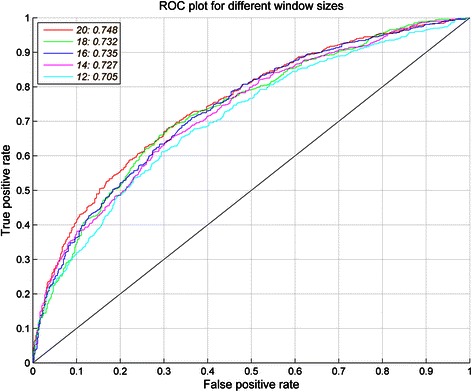
ROC curves of APCpred on BCI727 dataset for different window-sizes using fivefold cross-validation. The largest AUC is 0.748 at window size of 20.

We used the dataset BCI727 to evaluate the performances of APCpred. First, the epitope and non-epitope sequence features were generated from the peptide sequence using APC (*I = 3*). Then, the noise APC was removed by feature selection using *t*-test (*p* < 0.5) on training dataset. The dimensional reduced APC were used for SVMs trainings and model evaluation. The performance of APCpred at different window lengths (12, 14, 16, 18, 20) on the BCI727 dataset was shown in Table 3, which indicated that the best performance was at the window size 20 with AUC = 0.748 and accuracy (Acc) = 68.43%. ROC plot for different window sizes was shown in Figure 3.

**Table 3:** **Performances of APCpred on BCI727 dataset at different window sizes using fivefold cross-validation**

Window Sizes	Acc(%)	S_en_(%)	S_pe_(%)	MCC	AUC
12	65.68	65.48	65.89	0.314	0.705
14	66.30	66.58	66.02	0.326	0.727
16	67.13	67.68	66.58	0.343	0.735
18	68.23	69.05	67.40	0.365	0.732
20	68.43*	69.74	67.13	0.369	0.748

* The bold denotes the largest accuracy (A_CC_) value of the prediction.

### Assessing APCpred model building method using different datasets

In Chen’s report [11], AAP propensity scale was used in combination with a support vector machine (SVM) to construct a model which achieved optimal accuracy of 71.09% on Chen872 using fivefold cross-validation at window size 20. Further, they combined the AAP scale and five amino acid propensity (AP) scales using the SVM classifier in order to improve the prediction accuracy and achieved Acc of 72.54%.

In Wee’s report [13], the method Bayesb only achieved an accuracy of 68.50% and AUC of 0.74 on Chen’s dataset. In this study, we used fivefold cross-validation on the Chen872 dataset to compare APCpred (*I* = 3 and *p* < 0.5) with Bayesb, Chen’s AAP and the combination method of AAP and AP (Table 4). The result showed that APCpred method achieved better performance with AUC = 0.809 and Acc = 72.94% comparing to Bayesb (Acc = 68.50%) and Chen’s two methods (Acc = 71.09% and 72.54%).

**Table 4:** **Performances of APCpred, Bayesb, AAP, and the combination method of AAP and AP models on testing with Chen872 dataset using fivefold cross-validation**

Methods	A_CC_(%)	S_en_(%)	S_pe_(%)	M_CC_	AUC
APCpred	**72.94**	69.95	75.92	0.460	0.809
Bayesb	68.50	70.00	67.00	-	0.74
AAP	71.09	60.87	75.36	0.366	-
AAP + AP	72.54	63.56	76.48	0.404	-

“-” denotes unknown information.

“AAP + AP” is Chen’s combination method of AAP and five APs.

The bold denotes the largest A_CC_ value of the prediction.

We further evaluated the APCpred (*I* = 3 and *p* < 0.5) performance with ABC16 and Blind387 datasets. In the previous study, BCpred and AAP_BCpred_ had a comparison with ABCpred [12]. BCpred and AAP_BCpred_ were proven to outperform over ABCpred on the fivefold cross-validation of ABC16, but both methods failed in improving the prediction of the independent dataset Blind387. Using publicly available benchmark datasets, we were also able to compare APCpred with ABCpred, BCpred and AAP_BCpred_. First, we tested fivefold cross-validation on ABCP16 dataset to compare APCpred with ABCpred, BCpred and AAP_BCpred_, the result were summarized in Table 5. In terms of overall accuracy, the performance of APCpred was more accurate than ABCpred, but less accurate than BCpred and AAP_BCpred_ on fivefold cross-validation of ABC16 dataset. However, in terms of overall AUC values, AUC of APCpred was only less than BCpred (ABCpred AUC was unknown). These results showed that APCpred also improved the performance of fivefold cross-validation on ABC16 compared with ABCpred.

**Table 5:** **Performances of APCpred, ABCpred, BCpred, and AAP**_**BCPred**_ **on testing with ABC16 dataset using fivefold cross-validation**

Methods	A_CC_(%)	S_en_(%)	S_pe_(%)	M_CC_	AUC
APCpred	73.00	65.14	80.86	0.466	0.794
ABCpred	65.93	67.14	64.71	0.319	-
BCPred	**74.57**	70.14	79.0	0.493	0.801
AAP_BCPred_	73.14	50.17	95.57	0.518	0.782

“-” denotes unknown information.

The bold denotes the largest Acc value of the prediction.

The classifier built from ABC16 dataset was used to predict the liner B-cell epitopes from an independent dataset for validation. The prediction accuracy was then used to compare APCpred (*I* = 3 and *p* < 0.5) with other current prediction methods. The performances of the four classifiers trained with ABC16 dataset, and then tested with the independent dataset Blind387. The results were summarized in Table 6. In this case, APCpred outperformed the other three methods, the improvement were *6/387* for ABCpred, *8/387* for BCpred, and *13/387* for AAP_BCpred_. In summary, the performance of APCpred model demonstrated more accurate results than ABCpred, BCpred and AAP_BCpred_ models in terms of prediction accuracy, AUC, and Mcc on the independent test dataset.

**Table 6:** **Comparison of Performances among APCpred, ABCpred, BCpred, and AAP**_**BCPred**_ **models**

Methods	A_CC_(%)	S_en_(%)	S_pe_(%)	M_CC_	AUC
APCpred	**67.96**	56.15	79.00	0.362	0.748
ABCpred	66.41	71.66	61.50	*0.333	*0.736
BCpred	65.89	66.31	65.50	0.318	0.699
AAP_BCPred_	64.60	64.17	65.00	0.292	0.689

The four classifiers were trained using ABC16 dataset and evaluated using the third dataset of Blind287.

“*” denotes the information was obtained on online prediction of ABCpred with the third dataset though an automatic program script.

The bold denotes the largest A_CC_ value of the prediction.

Finally, we trained APCpred (*I* = 3 and *p* < 0.5) model on Lbtope_Confirm dataset [14] to test the variable length linear B-cell epitopes on FBC934 dataset, the result was summarized in Table 7. The APCpred accuracy is 55.09%, which is better than 52.66% from the model LBtope. Therefore, APCpred also improved the prediction on this dataset.

**Table 7:** **Comparison of performances between APCpred and LBtope on FBC934 dataset**

Methods	Acc(%)	S_en_(%)	S_pe_(%)	Mcc
LBtope on FBC934 dataset (trained on Lbtope_Confirm dataset)	52.66	78.09	27.23	0.06
APCpred on FBC934 dataset (trained on Lbtope_Confirm dataset)	**55.09**	59.31	50.86	0.10

Both models were trained on Lbtope_Confirm dataset.

The bold denotes the largest Acc value of the prediction.

## Discussion

B-cell linear epitopes are short sequences on the antigenic proteins, which contain structure characters to exposure themselves to antibodies, and easily bind to the antibodies, even if they are disengaged from the source proteins. In order to have antigenic functions, epitope sequences must be different from the random sequences generated from Swiss-prot database. We believe that B-cell linear epitopes sequences must fold into a stable structure to show the sequences’ information for being bound to antibodies. We propose that the amino acid anchoring pairs play important roles in stabilizing folding of epitopes structure by producing the force for folding in three-dimensional spaces. Thus, in this paper, we studied the roles of amino acid pairs in prediction of B-cell linear epitopes. Since it has been reported that 86.7% epitopes’ length was at most 20 amino acids in Bcipep database [12], during dealing with the large variability in the length of the epitopes, we chose to fix length of epitopes with lengths ranging from 12 to 20 peptides in the method of El-Manzalawy [12] and Saha [10], instead of windows of five or seven amino acids at the center of a linear epitope as Parker [5] and Karplus [6] did. The existing B-cell linear-epitope finding methods are far less than optimal or may only find part of epitope sequences, which may indicate that the prediction methods based on composition of amino acids properties might be better than the prediction methods based on amino acids exact positions.

Based on the APC in window of amino acids and feature selection, we explored a novel method, APCpred, for prediction of linear B-cell epitopes on primary amino acid sequences. We used BCI727 dataset to find the optimal parameters of APCpred (*I* = 3 and *p* < 0.5) by fivefold-cross validation. Our experiments using five cross-validation on the homology-reduced dataset of 727 non-epitopes, BCI727, showed that APCpred achieved max AUC = 0.748 and A_CC_ = 68.43%. It is interesting to find that the *I* = 3 (*i* = 0, 1, 2, 3) is identical to α-helix in protein second structure of which every backbone N-H group donates a hydrogen bond to the backbone C = O group of the amino acid four residues earlier (i + 4 to i hydrogen bonding. Thus, the number of interval amino acid is 3) [23]. This kind of arrangements maintains a regular and stable protein structure.

Using the other five datasets, we compared APCpred with several exiting methods, and the results demonstrated that the APCpred method improved the prediction of B-cell linear epitopes both in fivefold cross-validation of training dataset and in blind testing of validation datasets. In comparisons with Bayesb, Chen’s AAP and combination method of AAP and AP, APCpred achieved the best prediction accuracy on fivefold cross-validation. In another comparison, although BCpred and AAP_BCpred_ achieved higher accuracy than APCpred on fivefold cross-validation, both models showed less impressive accuracies than APCpred in prediction of blind dataset. Based on the comparison of APCpred and LBtope, APCpred method was also shown to be better than LBtope method. Therefore, we believe APC is a promising feature encoding method for improving prediction of linear B-cell epitopes.

Accurate prediction of linear B-cell epitopes requires effective features encoding, features selection and proper classifying methods. So far existing models remain suboptimal in these aspects. In this study, we tried to combine features encoding method APC, feature selection (*t*-test) and SVM to improve the prediction of linear B-cell epitopes. Our results demonstrated that the sequences of amino acid anchoring pair can capture the sequence feature pattern, and the classifying ability can be enhanced by feature selection. Using APC and the feature selection’s enhancement, APCpred model showed improvements over the current models, which may provide a novel method in predicting liner B-cell epitopes.

Based on our results in this study, we developed APCpred by Perl and PHP, and an online web server for predicting linear B-cell epitopes. It is freely available at http://ccb.bmi.ac.cn/APCpred/.

## Abbreviations

APC: Anchoring pair composition
SVM: Support vector machine
APCpred: A prediction model for linear B-cell epitopes based on the combination of APC and SVM
AP: Acid propensity
AAP: Amino acid pairs
Mcc: Matthews correlation coefficient
Acc: Accuracy
